# Interaction mechanism and intervention strategy between metabolic dysfunction-associated steatotic liver disease and intestinal microbiota

**DOI:** 10.3389/fmicb.2025.1597995

**Published:** 2025-09-18

**Authors:** Wentai Yang, Qing Jin, Dewang Xiao, Xiang Li, Defa Huang

**Affiliations:** ^1^Department of Gastroenterology, The First Affiliated Hospital of Gannan Medical University, Ganzhou, China; ^2^The First Clinical Medical College of Gannan Medical University, Ganzhou, China; ^3^Laboratory Medicine, The First Affiliated Hospital of Gannan Medical University, Ganzhou, China

**Keywords:** metabolic dysfunction-associated steatotic liver disease, gut microbiota, gut-liver axis, microbial metabolites, probiotics, prebiotics, fecal microbiota transplantation

## Abstract

The interaction between metabolic dysfunction-associated seatotic liver disease (MASLD) and gut microbiota regulates hepatic metabolic homeostasis through the gut-liver axis, and its mechanisms involve intestinal dysbiosis (decreased *bacteroidetes*, increased ratio of *firmicutes*/*proteobacteria*), bile acid metabolism reprogramming (secondary bile acids inhibit FXR signaling), short-chain fatty acid (SCFAs) deficiency, and endotoxin-mediated inflammatory activation (TLR4/NF-κB pathway). Among the intervention strategies, probiotics (such as *Bifidobacteria*) improved inflammation by regulating microbiota structure and intestinal barrier function, prebiotics such as resistant starch enriched butyric acid-producing bacteria and reduced liver lipid deposition, fecal microbiota transplantation (FMT) could remodel the microbiota but needed to optimize safety, restricted fructose intake and Mediterranean diet reduced liver damage by regulating microbiota metabolism, and metabolic surgery improved fibrosis through microbiota remodeling and bile acid signaling. In the future, it is necessary to combine multi-omics technology to analyze the microbiota-host interaction network, develop precision therapies such as phage targeted clearance or engineering bacterial delivery of metabolites, and promote the clinical transformation of personalized intervention programs.

## 1 Introduction

Metabolic dysfunction-associated steatotic liver disease (MASLD) is a global epidemic that affects approximately 25% of the adult population and is strongly associated with metabolic disorders such as obesity, insulin resistance, and type 2 diabetes (Kuang et al., 2023). In recent years, the interaction mechanism between gut microbiota and host metabolism through the gut-liver axis has gradually become the focus of research. Dysbiosis of the gut microbiosis can affect hepatic metabolic homeostasis through a variety of pathways, including bile acid metabolism reprogramming, abnormal production of short-chain fatty acids, and endotoxin-mediated inflammatory responses (Abenavoli et al., 2021). For example, gut microbiota-modified bile acids have been found to be inversely correlated with liver fat content (Kuang et al., 2023), while a reduction in the microbiota-derived tryptophan metabolite indole-3-acetic acid may exacerbate liver lipid deposition (Aron-Wisnewsky et al., 2020b). Animal experiments have further confirmed that dietary fiber can significantly improve inflammation and fibrosis in nonalcoholic steatohepatitis (NASH) by modulating microbiota metabolites such as 3-succinylcholic acid (Wei et al., 2023).

Notably, disruption of intestinal barrier function leads to translocation of microbial-associated molecular patterns to the liver, activating the Toll-like receptor pathway and inducing insulin resistance and hepatocyte damage (Scorletti et al., 2020). Clinical studies have revealed characteristic changes in the gut microbiota of patients with MASLD, including changes in the abundance of *Ruminococcaceae* and *Bifidobacterium*, which correlate with disease severity and fibrosis progression (Aron-Wisnewsky et al., 2020a). However, there is still heterogeneity in existing studies, such as the effects of age, gender, and dietary patterns on microbiota-host interactions in different populations that have not been fully elucidated (Aron-Wisnewsky et al., 2020a).

In terms of intervention strategies, precision therapy targeting intestinal microbiota has shown broad prospects. Clinical randomized controlled trials have shown that synbiotics (probiotics combined with prebiotics) can regulate microbiota composition but fail to significantly reduce liver fat content (Ye et al., 2024), while resistant starch has shown unique advantages in improving hepatic steatosis by enriching butyrate-producing bacteria and reducing branched-chain amino acid levels (Ni et al., 2023). In addition, specific probiotic strains such as *Bifidobacterium pseudolongum* inhibit liver cancer-related signaling pathways by secreting acetate, providing a new idea for the prevention of MASLD-related liver cancer (Leung et al., 2022). However, the role of fungal and virome in MASLD remains to be further explored, for example, the association between Candida and systemic immune responses suggests that fungal-host interactions may influence disease progression (Zheng et al., 2025).

This review systematically reviews the key molecular mechanisms of the interaction between MASLD and intestinal microbiota, evaluates the clinical translation potential of existing intervention strategies, and explores the application prospects of microbiome-targeted therapy in personalized medicine, aiming to provide a theoretical basis for the development of new diagnosis and treatment protocols based on the regulation of the gut-liver axis.

## 2 Mechanism of interaction between MASLD and gut microbiota

There is a complex bidirectional regulatory network between the pathogenesis of Metabolic Dysfunction-Associated Steatotic Liver Disease (MASLD) and the intestinal microbiota, which interact with liver metabolic homeostasis through intestinal barrier function, microbial metabolites, immune inflammatory pathways, and bile acid metabolism. We will systematically elucidate the core mechanism of intestinal microbiota regulating MASLD through the “gut-liver axis,” and discuss its molecular basis and clinical significance based on the latest research progress.

### 2.1 Pathological association between intestinal dysbiosis and MASLD

#### 2.1.1 Characteristic changes in microbiota composition and diversity

The pathological state of MASLD may have an impact on the composition and function of the intestinal microbiota. The study found that patients with MASLD had a decreased diversity of gut microbiota, a decrease in beneficial bacteria, and an increase in harmful bacteria (Sutanto et al., 2025). For example, in a mouse model of MASLD, changes in gut microbiota are closely related to liver lipid metabolism disorders and inflammatory responses (Hu Y. et al., 2025). Some specific gut bacteria, such as *Akkermansia muciniphila*, play an important role in MASLD. MASLD mice were given intragastric *A. muciniphila* reduces hepatic lipid accumulation and accelerates hepatic regeneration, possibly through the regulation of the tricarboxylic acid (TCA) cycle (Hu Y. et al., 2025). A number of clinical studies have shown that the α diversity of intestinal microbiota in MASLD patients is significantly reduced, and the microbiota structure presents characteristic disorders. A cohort study of 110 morbidly obese patients found that patients with MASLD had reduced abundance of *bacteroidetes* and an increased ratio of *Firmicutes* to *Proteobacteria*, an imbalance that was positively correlated with the severity of hepatic steatosis (Ji et al., 2024). Notably, excessive proliferation of specific genera such as *Prevotella* and *Ruminococcus* is strongly associated with elevated levels of inflammatory markers in the liver (e.g., ALT, CRP) (Iino et al., 2019). In contrast, butyrate-producing *Roseburia* and *Faecalibacterium prausnitzii* were significantly reduced in MASLD patients, suggesting that impaired microbiota metabolism may exacerbate intrahepatic lipid deposition through short-chain fatty acid deficiency (SCFAs) (Zhang et al., 2021).

#### 2.1.2 Metabolic output mechanism of dysbiosis

The gut microbiota directly or indirectly regulates hepatic lipid metabolism through metabolites. First, short-chain fatty acids (SCFAs) such as acetic acid, propionic acid, and butyric acid produced by the fermentation of dietary fiber by the microbiota can inhibit hepatic new lipogenesis and promote fatty acid oxidation by activating G protein-coupled receptors (GPR41/43)(Schoeler et al., 2023). However, the reduction of SCFAs-producing microflora in MASLD patients leads to impairment of this protective pathway (Yang et al., 2023). Second, indole derivatives produced by the metabolism of aromatic amino acids by the gut microbiota can activate the hepatocyte nuclear receptor FXR and inhibit the expression of the lipid synthase SREBP-1c, but its levels are significantly reduced in MASLD patients (Rao et al., 2021). In addition, bacterial-derived endotoxins enter the liver through the portal vein, activating the TLR4/NF-κB pathway and inducing intrahepatic inflammatory response and insulin resistance (Baffy, 2019). Intestinal microbiota-derived metabolites can participate in the inflammatory response of MASLD by post-translational modification of host proteins, affecting immune response and liver metabolism (Ajith and Anita, 2025). Patients with MASLD have an increase in ethanol-producing bacteria in the gut, resulting in an increase in endogenous ethanol, which induces oxidative stress and fat deposition in hepatocytes (Lanthier and Delzenne, 2022).

### 2.2 The core regulatory mechanism of the gut-liver axis

#### 2.2.1 Intestinal barrier dysfunction and metabolite leakage

**Figure 1:**
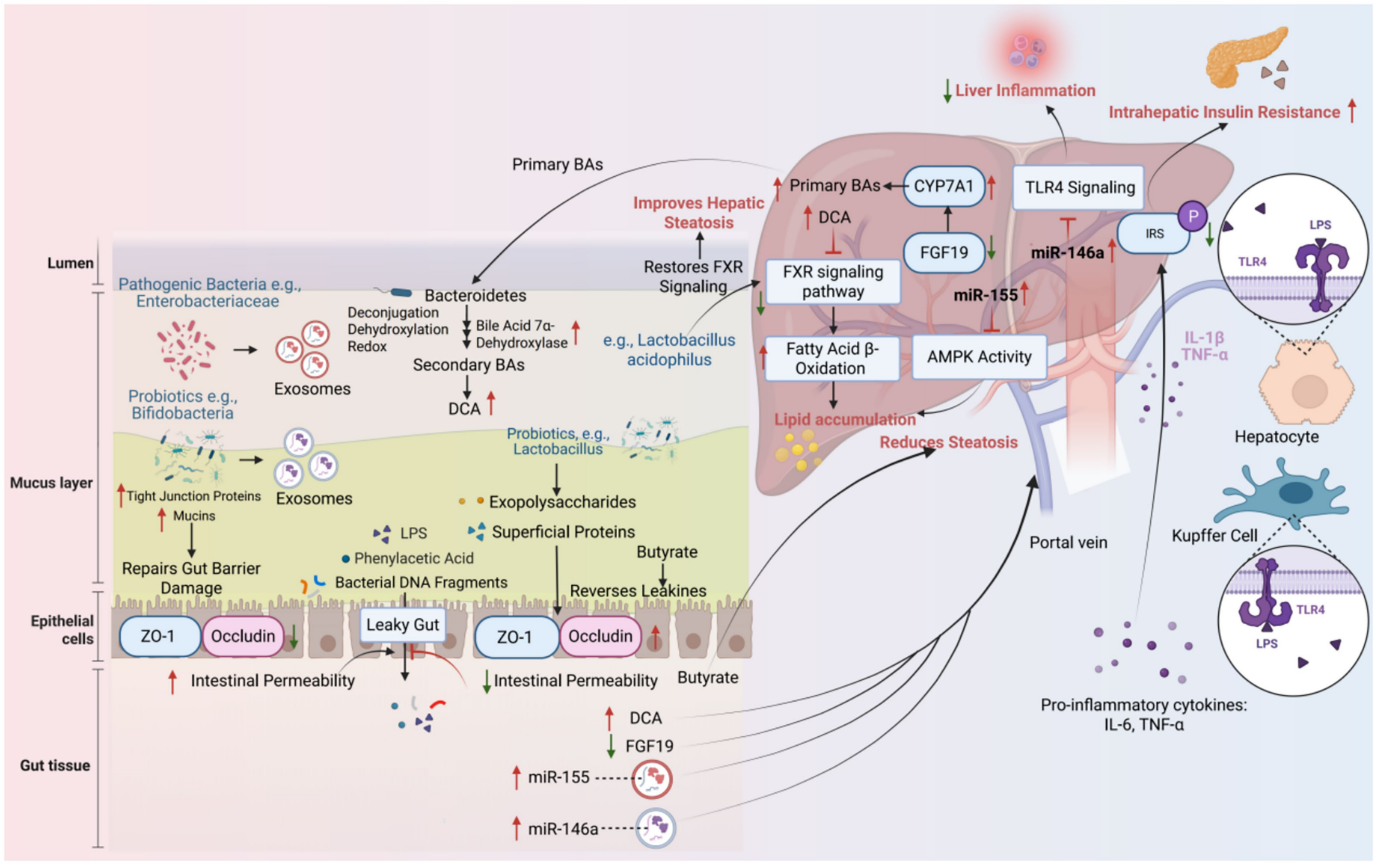
The core regulatory mechanism of the gut-liver axis.

The integrity of the intestinal barrier in patients with MASLD is impaired, which is manifested by down-regulation of the expression of tight junction proteins (such as ZO-1 and occludin) and a decrease in mucus layer thickness, resulting in the phenomenon of “leaky gut” (Baffy, 2019). This increased permeability promotes microbiota metabolites (e.g., LPS, phenylacetic acid) and bacterial DNA fragments to enter the liver through the portal vein circulation. Among them, LPS binds to TLR4 on the surface of hepatocytes and activates Kupffer cells to release pro-inflammatory factors (IL-6, TNF-α), which further inhibits insulin receptor substrate (IRS) phosphorylation and exacerbates intrahepatic insulin resistance (Hu S. et al., 2025) (Figure 1). Animal experiments have demonstrated that transplantation of fecal microbiota in MASLD patients with germ-free mice resulted in a significant increase in intestinal permeability accompanied by aggravation of hepatic steatosis, a phenotype that can be reversed by butyrate supplementation (Yang et al., 2023). The exopolysaccharides and superficial proteins produced by certain probiotics (e.g., *Lactobacillus*) in the biofilm state can significantly enhance the intestinal barrier function, upregulate the expression of tight junction proteins (e.g., ZO-1, Occludin), reduce intestinal permeability, thereby inhibiting toxins such as lipopolysaccharide (LPS) into the bloodstream, and reducing liver inflammation and steatosis (Li et al., 2024; Jo et al., 2024).

#### 2.2.2 Regulation of microbiota in bile acid metabolism

The intestinal microbiota modifies primary bile acids through debinding, dehydroxylation, and redox reactions to produce secondary bile acids (eg, deoxycholic acid DCA, lithocholic acid LCA). In MASLD patients, *Bacteroidetes* activity against bile acid 7α-dehydroxylase is enhanced, resulting in elevated levels of DCA, which impairs the β oxidative capacity of fatty acids in the liver by inhibiting the FXR signaling pathway (Baffy, 2019). In addition, FXR signaling inhibition leads to a decrease in fibroblast growth factor 19 (FGF19) secretion, which is unable to effectively inhibit intrahepatic CYP7A1-mediated bile acid synthesis, forming a vicious circle (Schoeler et al., 2023). Notably, specific probiotics, such as *Lactobacillus acidophilus*, can improve hepatic steatosis in mice by restoring FXR signaling, suggesting that the microbiota-bile acid axis is a potential therapeutic target (Jin et al., 2024).

#### 2.2.3 Exosome-mediated microbiota-hepatocyte communication

Recent studies have found that the intestinal microbiota can release exosomes containing miRNA, proteins and metabolites, and directly regulate hepatocyte metabolism through the portal vein. For example, miR-155 carried by exosomes of pathogenic bacteria (e.g., *Enterobacteriaceae*) enriched in the gut of patients with MASLD inhibits hepatocyte AMPK activity and promotes lipid accumulation (Zhang et al., 2022). In contrast, miR-146a in the exosomes of probiotics (e.g., *Bifidobacteria*) can target inhibition of TLR4 signaling and reduce liver inflammation (Craven et al., 2020). This finding provides a new idea for precise intervention based on microbiota exosomes. In addition, some studies have found that exosome-derived microRNAs can indirectly improve liver metabolism by enhancing the expression of tight junction proteins and mucins, repairing intestinal barrier damage (Liu et al., 2025).

### 2.3 Cross-linking of immune inflammatory mechanisms

#### 2.3.1 Activation of the innate immune system

Dysbiosis of the gut microbiosis activates the innate immune response in the liver through pattern recognition receptors (PRRs). In patients with MASLD, microbiota-derived peptidoglycan (PGN) binds to the NOD1 receptor and triggers hepatic stellate cells (HSCs) to secrete IL-17A, promoting neutrophil infiltration and liver fibrosis (Hu S. et al., 2025). At the same time, TLR9 recognizes bacterial CpG DNA, activates NLRP3 inflammasome, promotes IL-1β release, and accelerates hepatocyte apoptosis and steatosis (Tilg et al., 2021). Clinicopathological analysis showed that the expression level of NLRP3 in liver tissue of MASLD patients was negatively correlated with the microbiota diversity index (Moon et al., 2022).

#### 2.3.2 Regulatory imbalance of adaptive immunity

The gut microbiota is involved in MASLD progression by regulating T cell differentiation. The decrease in *Clostridium* in the gut of MASLD patients leads to limited differentiation of regulatory T cells (Tregs) and an increased proportion of Th17 cells, exacerbating intrahepatic inflammation (Tilg et al., 2021). Animal experiments have shown that supplementation with butyrate-producing *P. prasusus* promotes Treg proliferation and inhibits Th17 responses, thereby alleviating liver damage (Jin et al., 2024). In addition, tryptophan derivatives, a microbiota metabolite (e.g., indole-3-propionic acid), regulates Th17/Treg balance by activating the aryl hydrocarbon receptor (AhR), but its levels are significantly reduced in MASLD patients (Rao et al., 2021).

### 2.4 Interaction of genetic susceptibility and environmental factors

#### 2.4.1 Effect of gene-microbiota interaction on MASLD phenotype

Genome-wide association studies (GWAS) have found that polymorphisms in MASLD-related genes (such as PNPLA3, TM6SF2) can affect the composition of intestinal microbiota. For example, individuals carrying the PNPLA3 I148M mutation have decreased abundance of *Akkermansia* in their gut and over proliferate Enterobacteriaceae, a microbiota characteristic associated with more severe liver fibrosis (Iino et al., 2019). Mechanistic studies have shown that PNPLA3 mutations lead to abnormal lipid droplet structure in hepatocytes, and the release of lipid toxic substances can alter the metabolism of intestinal microbiota and form a positive feedback loop (Zeybel et al., 2022).

#### 2.4.2 Metabolic regulation of diet-microbiota interactions

High-fat diet (HFD) exacerbates MASLD progression by altering microbiota structure. Clinical studies have shown that low saturated fatty acid (SFA) intake is positively associated with the abundance of butyrate-producing microbiota, while a high SFA diet promotes the proliferation of Vibrio desulfur, which produces hydrogen sulfide through sulfate reduction, inhibits mitochondrial β oxidation and induces oxidative stress in hepatocytes (Schoeler et al., 2023). In addition, dietary polyphenols (e.g., cyanidin-3-glucoside) enrich *Roseburia* and *Bifidobacterium*, and their metabolites inhibit hepatic lipid synthesis by activating the PPARα pathway (Yang et al., 2023).

### 2.5 Systems biology perspective of microbiota-host metabolic networks

The integration of multi-omics data showed that the genes related to lipopolysaccharide synthesis, aromatic amino acid metabolism and bile acid conversion were significantly enriched in the functional genome of the intestinal microbiota of MASLD patients, while the expression of carbohydrate active enzyme (CAZymes) gene family was down-regulated (Rao et al., 2021). Metabolomic analysis further revealed that patients with MASLD had elevated plasma levels of branched-chain amino acids (BCAAs) and phenylacetyl glutamine (PAGln), and these microbiota-derived metabolites exacerbated liver injury by inhibiting AMPK signaling and promoting oxidative stress (Zhang et al., 2021). Systems biology models suggest that the core nodes of the microbiota-host co-metabolic network (e.g., FXR, PPARγ, TLR4) can serve as hubs for multi-target interventions (Baffy, 2019).

The interaction mechanism between MASLD and intestinal microbiota involves a complex regulatory network of multiple levels and organs, and the core of the interaction mechanism is that microbiota metabolites affect liver lipid metabolism, inflammatory response and insulin sensitivity through the gut-liver axis. The in-depth analysis of the key nodes of this network will provide a theoretical basis for the development of precision treatment strategies based on microbiota regulation.

## 3 Intervention strategies for the interaction between MASLD and gut microbiota

The interaction mechanism between Metabolic Dysfunction-Associated Steatotic Liver Disease (MASLD) and gut microbiota provides an important direction for the development of novel intervention strategies. Based on the central role of intestinal microbiota in the pathological process of MASLD (such as bile acid metabolism disorder, endotoxemia, short-chain fatty acid imbalance, etc.), the current intervention methods for intestinal microbiota mainly include microbial targeted therapy, dietary modification, metabolic surgery, and drug target development. The mechanisms, clinical evidence, and limitations of these strategies are systematically described below (Table 1).

**Table 1:** Intervention strategies for the interaction between MASLD and gut microbiota.

Therapy classification	Specific method	Mechanism of action/effect	Limitations/risks	References
Microbial targeted therapy				
	Probiotics	Regulate the structure of intestinal flora, enhance barrier function, and inhibit colonization of pathogenic bacteria: Reduces endotoxemia (e.g., Bifidobacteria, Lactobacillus reduce LPS) Regulates bile acid metabolism (e.g., upregulation of CYP7A1 by *Lactobacillus rhamnosus*) Increases short-chain fatty acids (e.g., *Clostridium butyricum* activates PPARγ)	Efficacy is affected by baseline microbiota composition; The long-term effects of advanced NASH are yet to be verified	Mehal (2013), Jiao et al. (2018), Kuang et al. (2023), Aron-Wisnewsky et al. (2020b)
	Prebiotics	Selectively promotes the proliferation of beneficial bacteria (e.g., inulin, fructooligosaccharides): Increases SCFAs and regulates bile acid pools	High doses cause gastrointestinal distress; limited effect in advanced fibrosis	Lambert et al. (2015)
	Synbiotic preparations	Increase the diversity of the microflora	No significant improvement in liver fat content and fibrosis	Scorletti et al. (2020)
	Antibiotics (such as rifaximin)	Inhibits pathogenic bacteria and reduces endogenous ethanol levels	Long-term use leads to dysbiosis or drug resistance	Meijnikman et al. (2022)
	Fecal Microbiota Transplantation (FMT)	Remodeling of microbiota structure and improvement of steatosis (e.g., increasing the abundance of Akkermansia)	risk of transmission of infection (e.g., ESBL-producing *E. coli*); Individual differences affect long-term stability; Safety standardization needs to be improved; Immune-related adverse effects	Marchesi et al. (2016), Beyer et al. (2024), Chuang et al. (2023), Facchin et al. (2025), Wang et al. (2016), Massaro et al. (2020)
Dietary interventions				
	A low-choline diet	Choline supplementation or targeting choline lyase may improve lipid metabolism disorders	The microbiota degrades choline to produce atherosclerogenic TMA	Spencer et al. (2011)
	Fructose restriction (<25 g/day)	Reduces microbiota-mediated acetic acid production and inhibits hepatic fat synthesis	–	Zhao et al. (2020)
	Mediterranean/high-fiber diet	Increase the diversity of microflora, reduce endotoxin, and regulate bile acid metabolism	Dietary fiber fermentation to produce propionic acid reduces hepatic glucose output	Marchesi et al. (2016)
	N-3 polyunsaturated fatty acids	Anti-inflammatory, regulates lipid metabolism and microbial diversity, and improves liver steatosis and function	–	Spooner and Jump (2019), Alwayn et al. (2005), Nobili et al. (2016)
Metabolic surgery	Gastric bypass surgery	Remodeling the microbiota: Bacteroides/Firmicutes ratio↑, butyric acid-producing bacteria proliferation→SCFAs↑ Secondary bile acids (e.g., lithocholic acid) activate TGR5 → enhance mitochondrial function	–	Sinclair et al. (2018)
Drug development				
	FXR agonists (e.g., obeticholic acid)	Inhibits liver lipid synthesis and improves NASH fibrosis	May cause pruritus and hyperlipidemia	Jiao et al. (2018)
	PPARα/*δ* agonists (e.g., Elafibranor)	Promotes fatty acid oxidation and improves insulin sensitivity	–	Mehal (2013)

### 3.1 Microbial targeted therapy

Probiotics play a therapeutic role by regulating the structure of intestinal flora, enhancing intestinal barrier function and inhibiting colonization by pathogenic bacteria. Potential mechanisms include: reduction of endotoxemia, such as *Bifidobacterium* and *Lactobacillus* by competitively inhibiting the growth of gram-negative bacteria and reducing lipopolysaccharide (LPS) levels, thereby reducing liver inflammation and insulin resistance (Mehal, 2013); regulate bile acid metabolism, promote the conversion of primary bile acids to secondary bile acids, activate farnesoid X receptor (FXR) and G protein-coupled bile acid receptor (TGR5), and inhibit liver lipid synthesis (Jiao et al., 2018), for example, *Lactobacillus rhamnosus* GG can upregulate liver CYP7A1 expression to alleviate steatosis (Kuang et al., 2023); Modulation of short-chain fatty acids (SCFAs), such as *Clostridium butyricum*, increases intestinal butyric acid levels and activates intestinal epithelial cells PPARγ to enhance barrier integrity (Mehal, 2013). Clinical studies have shown that supplementation with compound probiotics for 12 weeks can significantly reduce liver fat content and inflammatory markers in patients with MASLD (Mehal, 2013), but the efficacy may be affected by the composition of the baseline microbiota, and the long-term effect on advanced nonalcoholic steatohepatitis (NASH) still needs to be verified (Aron-Wisnewsky et al., 2020b).

Prebiotics work by selectively promoting the proliferation of beneficial bacteria, such as inulin and fructooligosaccharides, which increase SCFAs production and modulate the bile acid pool (Lambert et al., 2015). Clinical evidence suggests that inulin supplementation for 24 weeks reduces liver fat content by 30% and improves insulin sensitivity, but high doses may cause gastrointestinal upset and have limited efficacy in advanced fibrosis (Lambert et al., 2015). Although synbiotics (a combination of probiotics and prebiotics) can increase microbiota diversity, clinical trials have shown that they have no significant effect on liver fat content and fibrosis, suggesting the need for longer-term intervention or combination therapy (Scorletti et al., 2020). Antibiotics (e.g., rifaximin) reduce endogenous ethanol levels by inhibiting pathogenic organisms, and short-term use may improve liver enzyme markers, but long-term use may lead to dysbiosis or resistance (Meijnikman et al., 2022).

Fecal microbial transplantation (FMT) improves liver steatosis by remodeling the microbiota structure, and preliminary trials have shown that it can increase the abundance of *Akkermansia* and reduce fat content by 15% (Marchesi et al., 2016), but its safety and standardization procedures still need to be improved (Aron-Wisnewsky et al., 2020b). There is a significant risk of infection transmission from FMT, such as transmission of broad-spectrum β-lactamase (ESBL) *E. coli* and Shiga toxin-producing *E. coli* (STEC), and these risks underscore the importance of rigorous donor screening, such as testing donors for infectious disease pathogens (Beyer et al., 2024; Chuang et al., 2023; Zellmer et al., 2021). Donor screening criteria include the exclusion of high-risk factors such as diabetes and cardiovascular events, but the screening process faces challenges, including the changing FDA regulatory framework and practical difficulties in laboratory operations (Rasmussen et al., 2024; Woodworth et al., 2017b; Woodworth et al., 2017a). In terms of long-term stability, studies have shown that the transplanted microbiota can be stable in the recipient for up to 2 years, and the effectiveness of frozen samples can be maintained at −80 °C for up to 12 months, but long-term maintenance may be affected by individual differences in microbial composition and intestinal environment, which may lead to uncertainty (Facchin et al., 2025; Wei et al., 2022; Kumar et al., 2017; Sahle et al., 2024). In terms of immunologic adverse effects, FMT may be associated with events such as ulcerative colitis, muscle weakness, especially in the treatment of immune-related diseases, such as cancer immune checkpoint inhibitor therapy-associated colitis, although most adverse effects are mild, with an incidence of about 17.4% (Wang et al., 2016, Massaro et al., 2020, Ding et al., 2019, Feng et al., 2023, Tan et al., 2022). The overall safety assessment showed that no serious events were reported for FMT in the short to medium term, but long-term safety data are still limited, especially in children and immunocompromised patients, and further research is needed on the global incidence of adverse events (Lee et al., 2024; Rapoport et al., 2022; Wang et al., 2022). Regulatory challenges include a lack of standardization, such as differences in donor selection criteria, sample handling methods, and clinical protocols, which have hindered the widespread application of FMT in areas such as inflammatory bowel disease (IBD) (Fanizzi et al., 2024; Liu et al., 2024; Sutanto et al., 2025). Future studies should explore strategies to optimize microbiota stability, such as using probiotic pre-treatment with donated samples, to enhance efficacy (Sutanto et al., 2025).

### 3.2 Dietary interventions

Dietary strategies ameliorate MASLD by modulating microbiota-host metabolism interactions. A low-choline diet is associated with the degradation of choline by the microbiota to the production of atherosclerotic trimethylamine (TMA), and choline supplementation or targeting choline lyase may improve lipid metabolism disorders (Spencer et al., 2011). Restriction of fructose intake (<25 g/day) reduces microbiota-mediated acetic acid production and inhibits hepatic new fat synthesis (Zhao et al., 2020). The Mediterranean diet and the high-fiber diet play a role by increasing microbiota diversity, decreasing endotoxin levels, and regulating bile acid metabolism, with dietary fiber fermented by the microbiota to produce propionic acid reducing hepatic glucose export (Marchesi et al., 2016). Intake of N-3 polyunsaturated fatty acids can improve hepatic steatosis and liver function through anti-inflammatory and modulation of lipid metabolism and intestinal microbiota diversity (Spooner and Jump, 2019; Alwayn et al., 2005; Nobili et al., 2016). Furthermore, high intake of n-3 PUFAs before conception may have a protective effect on the development of obesity-related NAFLD, and this influence has the characteristic of intergenerational transmission (Shi et al., 2024). Theabrownin (TB) is the main bioactive component in pu-erh tea, and it has been found that TB reduces the levels of ceramides by inhibiting the intestinal FXR-Cer synthase axis, and thus ameliorates hepatic steatosis, inflammation and oxidative stress (Wang et al., 2024). An isoleucine-restricted diet effectively prevented HFD-induced NAFLD and metabolic disorders by modulating intestinal flora, reducing LPS production, inhibiting the TLR4/NF-κB inflammatory pathway, and improving insulin resistance (Zhou et al., 2024).

### 3.3 Metabolic surgery

Metabolic surgeries such as Roux-en-Y gastric bypass ameliorate MASLD by remodeling microbiota and modulating bile acid signaling. An increase in the postoperative *bacteroidetes/firmicutes* ratio and the proliferation of butyric acid-producing bacteria increased the level of SCFAs, while activation of TGR5 by secondary bile acids (e.g., lithocholic acid) enhanced hepatocyte mitochondrial function (Sinclair et al., 2018). Clinical studies have shown a 60% reduction in liver fat content and a significant improvement in fibrosis scores 1 year after surgery (Sinclair et al., 2018).

### 3.4 Drug target development

Significant progress has been made in the development of drugs targeting pathways related to the gut microbiota. FXR agonists (e.g., obeticholic acid) ameliorate NASH fibrosis by inhibiting hepatic lipid synthesis but may trigger pruritus and hyperlipidemia (Jiao et al., 2018). PPARα/*δ* agonists (e.g., Elafibranor) work by promoting fatty acid oxidation and improving insulin sensitivity (Mehal, 2013).

## 4 Limitations and prospects

The current intervention strategies face challenges such as individualized treatment needs, optimization of combination therapies, and long-term safety assessment. Precise typing based on microbiota characteristics (e.g., intestinal type classification) may improve efficacy (Spencer et al., 2011), while the synergistic effect of probiotics in combination with FXR agonists or dietary interventions needs to be further validated (Lambert et al., 2015). In addition, the risk of resistance and the immune impact of antibiotics or FMT need to be critically evaluated (Aron-Wisnewsky et al., 2020b). In the future, it is necessary to analyze the microbiota-host interaction network by combining multi-omics technology, and verify the feasibility of personalized protocols through large-scale clinical trials.

Significant progress has been made in the study of the interaction mechanism between metabolism-related fatty liver disease (MASLD) and intestinal microbiota, but it still needs to be explored in many aspects in the future.

Refinement of mechanism research: At present, the molecular mechanism of intestinal microbiota affecting liver lipid metabolism and inflammation through bile acid metabolism, short-chain fatty acids, endogenous ethanol and other pathways still needs to be further analyzed. For example, the mechanism by which microbiota-modified bile acids improve lipid deposition by activating hepatocyte CYP7B1 and PPARα signaling pathways (Kuang et al., 2023), and pathway in which specific microbiota (e.g., *Odoribacteraceae*) regulates the hepatic immune microenvironment through metabolites, need to be validated in more clinical cohorts (Miyamoto et al., 2024). In addition, the mechanism of how metabolites in the microbiota-gut-liver axis, such as acetic acid, promote hepatic lipid synthesis through ACSS2 still needs to be further explored (Zhao et al., 2020).

Development of individualized intervention strategies: Individual differences in gut microbiota significantly affect the disease phenotype and treatment response of MASLD. For example, changes in the abundance of *Gammaproteobacteria* and *Erysipelotrichi* in the gut of different individuals under a choline-deficient diet are directly related to the degree of hepatic fat accumulation (Spencer et al., 2011), suggesting that individualized diets or probiotic/prebiotic regimens based on microbiota characteristics will be needed in the future. Integrated analysis of multi-omics technologies (e.g., metagenomics, metabolomics) will facilitate precise typing and targeted interventions (Aron-Wisnewsky et al., 2020b).

Application of new microbial control technologies: The clinical efficacy of existing intervention methods (such as probiotics and synbiotic preparations) is controversial (Scorletti et al., 2020), and more efficient microbiota editing tools need to be explored in the future. For example, phage-targeted clearance of pro-inflammatory flora [e.g., Lactobacillaceae is associated with endogenous ethanol production (Meijnikman et al., 2022)] or delivery of specific metabolites by engineered bacteria may be new directions. In addition, the potential of fecal microbiota transplantation (FMT) in MASLD needs to be validated urgently (Mehal, 2013).

Integrative studies of cross-organ interaction networks: The association of MASLD with chronic kidney disease (CKD) suggests that microbiota metabolites may affect multi-organ function through systemic inflammation or oxidative stress (Byrne and Targher, 2020). In the future, it is necessary to combine single-cell sequencing and spatial transcriptome technology to elucidate how the microbiota regulates systemic metabolic disorders through the gut-liver-kidney axis (Musso et al., 2016).

The limitations of this review article are mainly reflected in the following aspects: first, the existing research on the interaction between MASLD and gut microbiota has significant technical problems and design biases, for example, when analyzing the diversity of gut microbiota, the reliability of the conclusions may be affected due to the limitations of technical methods (such as sequencing depth or sample processing bias), which limit our understanding of the precise role of gut microbiota in MASLD pathology (Gil-Gómez et al., 2021); Second, the important evidence supporting the role of gut microbiota in the development of MASLD mainly comes from animal experimental studies, while data based on human clinical studies are relatively scarce, and this dependence leads to uncertainties in translational applications, such as insufficient effectiveness in validating mechanisms such as bile acid metabolism or TLR4/NF-κB inflammatory pathways in human patients (Bakhshimoghaddam and Alizadeh, 2021); In addition, although intervention strategies (such as probiotics, prebiotics, or fecal microbiota transplantation) have shown potential efficacy, the existing literature shows that their clinical application still needs to optimize key parameters such as bacterial strain selection, treatment time, and dose determination, and the current lack of standardized protocols limits the replicability of large-scale clinical practice (Bakhshimoghaddam and Alizadeh, 2021). In terms of quantitatively assessing the association between gut microbiota and MASLD, there is a lack of systematic bibliometric analysis or multi-omics integration methods, which may ignore hot trends and knowledge gaps, and hinder the development of future precision interventions (Li et al., 2022). Finally, geographic factors (e.g., diet or lifestyle) and virome/fungal influences are not discussed in depth, and these limitations reflect the shortcomings of current research and highlight the need to strengthen human clinical trials, improve technical frameworks, and promote interdisciplinary integration.

## 5 Conclusion

The interaction mechanism between metabolism-related fatty liver disease and intestinal microbiota is complex and multi-layered, involving the direct regulation of hepatocyte lipid metabolism, immune cell function and intestinal barrier integrity by microbial biota metabolites. Available evidence suggests that imbalances in bile acid metabolism, endogenous ethanol production (Meijnikman et al., 2022), and abnormal short-chain fatty acid signaling (Zhao et al., 2020) are central components of microbiota-driven MASLD progression. In terms of intervention strategies, probiotics/prebiotics can regulate the structure of microbiota and improve hepatic steatosis (Marchesi et al., 2016), but the long-term efficacy is affected by individual microbiota heterogeneity and intervention timing (Scorletti et al., 2020); Targeting specific metabolic pathways, such as FXR agonists or PPARα activators (Kuang et al., 2023), may be more specific.

However, there are still limitations in current research: most of the mechanistic evidence comes from animal models, and clinical translation needs to overcome species differences; Randomized controlled trials of microbiota interventions were small and endpoints were inconsistent (Lambert et al., 2015); The interaction between microbiota and host genetics and diet has not been fully elucidated (Spencer et al., 2011). In the future, it is necessary to build a microbiota-host interaction network through multi-omics integration, organoid models, and artificial intelligence prediction, and promote the clinical practice of personalized intervention programs. Finally, an integrated management strategy targeting gut microbiota is expected to open up a new path for the prevention and treatment of MASLD (Aron-Wisnewsky et al., 2020b).
